# Heritability and Phenotypic Variation of Canine Hip Dysplasia Radiographic Traits in a Cohort of Australian German Shepherd Dogs

**DOI:** 10.1371/journal.pone.0039620

**Published:** 2012-06-27

**Authors:** Bethany J. Wilson, Frank W. Nicholas, John W. James, Claire M. Wade, Imke Tammen, Herman W. Raadsma, Kao Castle, Peter C. Thomson

**Affiliations:** Faculty of Veterinary Science, The University of Sydney, Sydney, New South Wales, Australia; University of Utah, United States of America

## Abstract

Canine Hip Dysplasia (CHD) is a common, painful and debilitating orthopaedic disorder of dogs with a partly genetic, multifactorial aetiology. Worldwide, potential breeding dogs are evaluated for CHD using radiographically based screening schemes such as the nine ordinally-scored British Veterinary Association Hip Traits (BVAHTs). The effectiveness of selective breeding based on screening results requires that a significant proportion of the phenotypic variation is caused by the presence of favourable alleles segregating in the population. This proportion, heritability, was measured in a cohort of 13,124 Australian German Shepherd Dogs born between 1976 and 2005, displaying phenotypic variation for BVAHTs, using ordinal, linear and binary mixed models fitted by a Restricted Maximum Likelihood method. Heritability estimates for the nine BVAHTs ranged from 0.14–0.24 (ordinal models), 0.14–0.25 (linear models) and 0.12–0.40 (binary models). Heritability for the summed BVAHT phenotype was 0.30±0.02. The presence of heritable variation demonstrates that selection based on BVAHTs has the potential to improve BVAHT scores in the population. Assuming a genetic correlation between BVAHT scores and CHD-related pain and dysfunction, the welfare of Australian German Shepherds can be improved by continuing to consider BVAHT scores in the selection of breeding dogs, but that as heritability values are only moderate in magnitude the accuracy, and effectiveness, of selection could be improved by the use of Estimated Breeding Values in preference to solely phenotype based selection of breeding animals.

## Introduction

Canine Hip Dysplasia (CHD) has been considered the most common musculoskeletal disease affecting both purebred dogs and their crossbreds. It is a multifactorial disease, with occurrence and severity modulated by many non-genetic factors and the action of genes at multiple loci in the dog genome, potentially including some major genes [1]–[5]. While hip structures develop to be grossly normal prenatally, in some dogs an intrinsic excess laxity or “looseness” in the joint causes abnormal joint forces once weight-bearing begins, resulting in misshaping of the cartilaginous matrix of the developing hip and repetitive microdamage to joint structures. Over time, this leads to the development of potentially severe osteoarthrtits [6]–[8]. CHD is a significant welfare problem, can result in significant disability and can lead to euthanasia on humane grounds.

Given the overwhelming evidence of additive genetic variation for liability to CHD, many selection schemes have been established worldwide to reduce the incidence (if dogs are classified as either affected or normal) or average severity of CHD in dog populations using various phenotypic assessments of hip laxity and morphology, mostly obtained by various radiographic techniques [9]. One such set of traits which has been used by the British Veterinary Association/Kennel Club Scheme (BVA/KC scheme) and later by the Australian Veterinary Association/Australian National Kennel Council (AVA/ANKC scheme), as well as the German Shepherd Dog Council of Australia Hip Dysplasia Breed Scheme (GSDCA scheme) and other schemes, is based on a set of hip phenotypes referred to here as the BVA hip traits (BVAHTs).

The BVAHTs are a collection of nine radiographic traits of a hip, collected from a radiograph of a skeletally mature dog, taken in an “extended hip” position, where the dog is placed in dorsal recumbency and hips are extended with legs held in parallel to each other and to the radiographic plate, with a slight internal rotation rendering the patellae directly above the stifle joint. The nature of these nine traits has been reported previously [10]–[12].The traits are all scored on a subjective seven-point ordinal scale (labelled 0–6) except Norberg angle (NORB), which is an objective quantitative trait (an angle measurement) that is collapsed into seven ordinal categories (with lower scores indicating higher angles); and Caudal Acetabular Edge (CaAE), which is scored on a subjective ordinal six-point scale (labelled 0–5). For each of these traits, 0 indicates a sound hip, and higher scores indicate increasing degrees of deterioration. Previous research on the genetic correlation between the BVAHTs of the right and left hip has provided estimates very close to 1.0, indicating that, in essence, the same genes modulate the formation and development of both hips and that variation between them is a result of non-genetic (environmental) variation experienced differently by each hip [12]–[15].

The effectiveness of phenotype-based selection schemes for a multifactorial disease is dependent upon the nature and extent of the variation that is seen in the population for this phenotype. The variation of the phenotype must be at least partly heritable and must also be genetically correlated to the selection goal, in this case improved health, function and welfare with respect to Canine Hip Dysplasia. In other words, dogs with better hip scores must both tend to have offspring with better hip scores (to some degree) and also to have better health, function and welfare (to some degree) than dogs with worse hip scores. If this is the case, it may be presumed that dogs with better hip scores will tend to have alleles which confer a health, function and welfare advantage, and that by transmitting these alleles to offspring they will similarly confer some degree of health, function and welfare advantage on their offspring. Preferentially selecting such dogs for breeding will tend to cause an increase in the frequency of advantageous alleles (and a decrease in the frequency of disadvantageous alleles), resulting in a favourable genetic trend and improvement in the hip dysplasia status of the population over generations. The other factor determining response to selection is the extent of variability in the trait undergoing selection. It is thus important to estimate the extent of variation and the heritability of the chosen phenotype in the population under selection, and to show that the chosen phenotype correlates to some extent with health, function and welfare.

Despite the operation of a hip dysplasia evaluation scheme in Australia for several decades, there is a disappointing paucity of studies evaluating the suitability and effectiveness of genetic control of hip dysplasia in Australian dog populations. Ideally, the extent of the additive genetic variation in hip dysplasia traits would be assessed and tracked temporally in all breed populations under selection in Australia, to ensure that favourable genetic trends are emerging over time and to monitor variation. Recent work by Lewis *et al*. [13], and earlier work by Wood *et al*. [16], Wood *et al*. [17] and Wood *et al*. [18], demonstrates substantial heritability and substantial phenotypic variation of BVAHTs in dog populations in the United Kingdom. Other studies [13], [15]–[24] have shown substantial heritability of different radiographic hip dysplasia phenotypes in various German Shepherd Dog populations (Table 1). While these results are encouraging, estimates of phenotypic variation and heritability should be obtained in Australian populations, as allele frequencies might be expected to vary between breed populations, and certainly between breeds, and over time. The correspondence between hip dysplasia phenotypes and hip dysplasia-related health, function and welfare is considerably more challenging to study, and perhaps because of this, there are regrettably few studies assessing this question. An important recent study [25] does demonstrate a relationship between insurance claims related to hip dysplasia and a hip dysplasia phenotype assessing similar features to the BVAHTs from a similar radiograph (although the phenotype is quantified quite differently).

**Table 1:** A sample of heritability estimates for British Veterinary Association Hip Traits in various breeds and heritability of other Canine Hip Dysplasia Phenotypes in German Shepherd Dogs.

Phenotype	Breed	Heritability	Standard Error	Method	Reference
**BVAHT-NORB**	Labrador Retriever	0.29	0.05	Regression	[16]
**BVAHT-NORB**	Gordon Setter	0.19	0.08	Regression	[18]
**BVAHT-NORB**	Labrador Retriever	0.37	0.03	REML LMM	[15]
**BVAHT-SUBL**	Labrador Retriever	0.26	0.04	Regression	[16]
**BVAHT-SUBL**	Gordon Setter	0.24	0.08	Regression	[18]
**BVAHT-SUBL**	Labrador Retriever	0.38	0.03	REML LMM	[15]
**BVAHT-CrAE**	Labrador Retriever	0.17	0.04	Regression	[16]
**BVAHT-CrAE**	Labrador Retriever	0.21	0.02	REML LMM	[15]
**BVAHT-DAE**	Labrador Retriever	0.16	0.06	Regression	[16]
**BVAHT-DAE**	Labrador Retriever	0.18	0.02	REML LMM	[15]
**BVAHT-CrEAR**	Labrador Retriever	0.07	0.05	Regression	[16]
**BVAHT-CrEAR**	Labrador Retriever	0.21	0.02	REML LMM	[15]
**BVAHT-AF**	Labrador Retriever	0.17	0.06	Regression	[16]
**BVAHT-AF**	Labrador Retriever	0.15	0.02	REML LMM	[15]
**BVAHT- CaAE**	Labrador Retriever	0.08	0.06	Regression	[16]
**BVAHT-CaAE**	Labrador Retriever	0.15	0.02	REML LMM	[15]
**BVAHT-FHNE**	Labrador Retriever	0.14	0.05	Regression	[16]
**BVAHT-FHNE**	Labrador Retriever	0.24	0.03	REML LMM	[15]
**BVAHT-FHR**	Labrador Retriever	0.15	0.05	Regression	[16]
**BVAHT-FHR**	Labrador Retriever	0.19	0.03	REML LMM	[15]
**BVAHT-Total**	Flat Coated Retriever	0.74	0.25	Regression	[17]
**BVAHT-Total**	Newfoundland	0.49	0.08	Regression	[17]
**BVAHT-Total**	Gordon Setter	0.20	0.10	Regression	[18]
**BVAHT-Total**	Labrador Retriever	0.34	0.02	Regression	[16]
**BVAHT-Total**	Labrador Retriever	0.35	0.02	REML LMM	[13]
**I-VI grading**	German Shepherd Dog	0.43–0.43	0.08	Least squares ANOVA	[20]
**1–5 (A-E)**	German Shepherd Dog	0.24–0.26	0.02–0.04	Bayesian LMM with animal model	[19]
**A-E**	German Shepherd Dog	0.31–0.35		REML LMM with animal model	[22]
**A-E**	German Shepherd Dog	0.15	0.02	Binary GLMM	[23]
**1–5**	German Shepherd Dog	0.56	0.011	REML LMM	[21]
**A-E**	German Shepherd Dog	0.254	0.013	Bayesian LMM with animal model	[24]

NORB = Norberg Angle, SUBL = Subluxation, CrAE = Cranial Acetabular Edge, DAE = Dorsal Acetabular Edge, CrEAR = Cranial Effective Acetabular Rim, AF = Acetabular Fossa, CaAE = Caudal Acetabular Edge, FHNE = Femoral Head and Neck Exostosis, FHR = Femoral Head Remodelling.

An Estimated Breeding Value (EBV) is the weighted combination of the scores of an individual and its relatives for the trait in question, for correlated traits and for molecular information; providing the best available prediction of the average performance of the offspring of that animal. Unless the heritability of the trait is very high (virtually all phenotypic variation arises from variation in genetic merit), EBVs will represent a better assessment of genetic merit than phenotypic scores alone. Therefore, if we determine BVAHTs to be heritable (but not of very high heritability), as has been reported in studies from other dog populations, this will be strong evidence that selection using EBVs should replace phenotype-based selection in this population.

In this paper we estimate the extent of phenotypic variation and the heritability of BVAHTs in a cohort of Australian-born German Shepherd Dogs. Given that BVAHTs are ordinal phenotypes, we report the use of a mixed-model multi-threshold ordinal logistic regression technique to estimate heritability. Further, we also fit models to the BVAHTs ignoring the ordinal nature of the data, using standard Restricted Maximum Likelihood (REML)/linear mixed model techniques, which is the typical strategy used in other studies. This dual approach allows us to investigate the extent to which ignoring the ordinal nature of the BVAHT data may affect such estimates.

## Materials and Methods

### Data

Sources of data were 1) data accumulated by Dr Malcolm Willis in the United Kingdom from records collected within the Australian Veterinary Association/Australian National Kennel Council (AVA/ANKC) scheme and the records of radiologists sent to him privately (kindly provided by the ANKC); 2) Data supplied by the German Shepherd Dog Council of Australia (GSDCA) hip dysplasia breed scheme; and 3) Pedigree information regarding Australian German Shepherd Dogs (GSDs) held by the ANKC and supplied to us with permission of the GSDCA by Dogs NSW. All data sets included all data available electronically at the time at which the records were obtained.

Australian records obtained from Dr Willis included records from about 10020 dogs, born between 1976 and 2005. Records obtained from the GSDCA scheme included records from approximately 6065 animals born between 1977 and 2006. There was considerable overlap between the two sets of data with respect to the dogs represented. Where more than one record was available for any unique dog, any identical duplicated records were deleted. Of the remaining **rare cases**, where phenotypes had been obtained at different times, only the record taken closest to the average age of radiography was retained. The data set included the animal’s name, pedigree information, year of birth, age at radiographic study, sex and scores for each of the eighteen BVAHTs. Any record that lacked any information was removed from the data set, unless this information could be inferred from the pedigree file. In the pedigree file, duplicate records were removed. Dogs were matched to the pedigree file using their registered names, date of birth and available pedigree information. Animals that were born overseas (as evidenced by an overseas-type registration number and lack of an Australian kennel prefix) were excluded from the phenotype file but were retained in the pedigree file.

The final data set comprised records from 13,124 (8,793 female, 4,331 male) GSDs born in Australia between 1976 and 2006. Completeness of the data set was investigated by matching scores against the pedigree file for Australian-born GSDs.

### Models

Three different types of single-trait models were used, namely (1) a “full” ordinal logistic model that treats the BVAHT data in a multi-threshold approach, with each threshold corresponding to a point on the ordinal scale; (2) a series of binary logistic models in which each model involves a single threshold at a different BVAHT ordinal point; and (3) a standard linear mixed model fitted to each of the 18 BVAHTs and to the summed BVAHT data, ignoring the ordinal nature of the data. Only single-trait models were conducted because the software used was not able to run multi-trait models assuming ordinal data. A previous paper [12] offered evidence which suggests that right and left scores for each BVAHT in Australian GSDs are two expressions of the same set of genes, with differences arising due to non-additive genetic and non-genetic causes, and could therefore be considered repeated measures. The alternatives to this approach are to select only a single hip from each dog (either systematically, such as taking the worst hip, or selecting a record at random) or to employ some measure of central tendency of the two measurements. The former approach needlessly halves the data set by removing meaningful information about each animal’s likely genetic merit. The latter approach, employing a measure of central tendency, requires finding a methodologically sound method of doing so. Taking the mean or median of the scores assumes linearity of scores that are truly ordinal, which is not readily methodologically justified. Given the evidence that right and left hip scores have a genetic correlation near one, the assumptions behind the repeatability model appear to be better supported by the available evidence and this method was therefore used in the present analysis. Modelling was undertaken using the “stand alone” version of ASReml 3 (VSN Intl., Hemel Hempstead UK).

#### 1) Ordinal (Multi-threshold) analysis

This model considers two scores (left and right) from each of n dogs. For a single observation in the data set, the model has the following form:
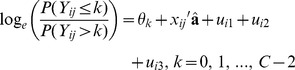
(1)where *Y_ij_* is the BVAHT score of the *i* ^th^ dog (*i*  = 1, …, *n*) on the *j* ^th^ side (*j*  = 1, 2), *C* is the number of points on the ordinal scale (*C* = 7 for all BVAHT except CaAE where *C* = 6). For each cut point, there is a separate “intercept” (θ*_k_*), with the constraint that θ_0_<θ_1_<…< θ*_C_*_–2_. **β** is a vector of *p* levels of fixed effects related to the vector of explanatory variables, **x** *_ij_*. The random effects for this model are *u_i_*_1_, a term for the dog’s breeding value; *u_i_*_2_, a term for the permanent environment effect of the dog linking left and right hand scores together; and *u_i_*_3_, a litter effect. This form of ordinal logistic regression is known as the proportional odds model [26].

When considered as a model for the vector of all 2*n* observations, the model can be expressed in matrix notation as
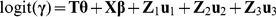
(2)where **γ**  = {γ*_ij_*} with 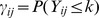, **T** is a 2*n*×(*C*–1) matrix with elements in column *k* taking values 1 if the score *Y_ij_* ≤ *k* and 0 otherwise, 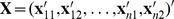 is a 2*n*× *p* matrix of predictor variables, the indicator matrices 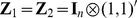 are of dimension 2*n*×*n*, and **Z**_3_ is of size 2*n*×*L* where *L* is the number of litters. The logit(⋅) function is defined as logit(γ)  = log*_e_*[γ/(1–γ)].

Fixed effects incorporating **β** include the sex of the dog (male or female), the age of the dog in months at the time of radiographic study, the year in which the dog was born, and left vs right hip. The random effects **u**_1_ are “animal model” additive genetic effects. The animal model is fitted by calculation of the numerator relationship matrix (NRM), a matrix of additive genetic relationships which contains information about the flow of genes through the population and information allowing for inbreeding. The permanent environmental effect associated with each dog is **u**_2_ and the effect associated with being from each litter is **u**_3._ The model also assumes that 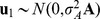 where 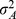 is the additive genetic variance, **A** is the NRM; and also that 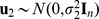 and 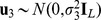.

This model is advantageous over linear methods as it recognises the ordinal nature of the data, rather than assuming that each cut point is evenly spaced along an underlying scale, and is advantageous over individual binary models as it does not require loss of information above and below a cut point. Therefore, for comparison among the different EBVs generated from the different models in this study, we treat these ordinal EBVs as the “gold standard” EBVs to which the others, either less methodologically correct, or calculated using less information, are compared. Due to the manner in which ASReml parameterises the ordinal model, it was necessary to multiply effects from the ASReml output by -1 to maintain correct associations and a scale in which a number above zero corresponds to an increase in average expected score of offspring (the undesirable direction) and a negative number corresponds to a favourable breeding value.

#### 2) Binary (Logistic) analysis

In addition to the multi-threshold ordinal analysis of the nine BVAHTs, modelling using the same fixed and random effects as described above was undertaken at each possible cut point, i.e. at each interval (on the ordinal scale) at which a threshold can be used to divide dogs into two classes: normal and affected. To accommodate the binary nature of these data, a logistic generalised linear mixed model (GLMM) was fitted to the data.

The seven-point scale of most of the traits offered a maximum of six possible cut points at which a hip could be classified as normal (0) or affected (1). For CaAE there were five possible cut points. Logistic regression analysis was attempted at each cut point. Each analysis makes fewer unsupported assumptions than the conventional linear mixed model LMM (see below) but involves loss of information consequential upon the pooling of all scores above and all scores below the cut point, e.g. at a cut point between 1 and 2, a score of 2 is awarded the same phenotype (1) as a score of 3, 4, 5 or 6. The form of the GLMM is
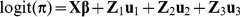
(3)where logit(π_i_ ) = log*_e_*[π*_i_*/(1–π*_i_* ), and π*_i_* is the probability that dog *i* has a score at or below the cut point. All the other terms in the model are as specified in [EQ2]. A separate logistic GLMM was fitted for each BVAHT× cut-point combination.

#### 3) Linear mixed models (LMM)

In this analysis, each of the nine BVAHTs was modelled by an LMM using the score as the phenotype. This strategy, which ignores the ordinal nature of the scoring system, was undertaken to compare EBVs with those obtained taking the ordinal nature into account, and to enable direct comparison with most other comparable studies. Scores were transformed logarithmically to attempt to correct positive skew in the distribution of the scores. While reduced, substantial skew remained for many traits. As the main role of this analysis was to compare results from a more correct ordinal analysis with a simpler analysis, more powerful transformations, while possible, were not attempted. Lewis *et al*. [15] performed analyses on untransformed scores after not finding a single transformation which was optimal for all nine BVAHTs and in order to simplify their analysis. For this study, the authors felt that the log-transformation for all traits to correct some, but not all, of the positive skew of each trait was the best compromise between methodological correctness and clarity. The model was of the form
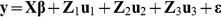
(4)where **y** is a vector of 2*n*  = 26,248 hips, and **ε** is a 2*n*×1 vector of random residual effects, where **ε**∼ N(**0**,σ^2^_ε_ **I** *_2n_*). All other terms are as defined in [EQ2] and [EQ3].

In addition to the analysis of individual BVAHTs, total hip scores (THS) were obtained by addition of the 18 BVAHT scores for each dog. This has been the standard trait used for selection in the GSDCA scheme and in all other schemes using BVAHTs. The scores were again logarithmically transformed. A linear mixed model was then fitted to the THS data of the form
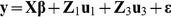
(5)where **y** now is a vector of *n*  = 13,124 THS observations, and **X** does not include a term for left versus right hip. The model does not include a permanent environment effect for the dog because there is now only one record for each dog in the data set, but does include a litter effect (**u**_3_).

#### 4) Heritability estimates

Heritability estimates were obtained by calculation of the proportion of the total variance explained by additive genetic variance. Heritability estimates were obtained for all analyses of all BVAHTs.

For ordinal and binary models, heritability for the trait on the underlying scale was calculated as σ^2^ *_A_* /( σ^2^ *_A_*+σ^2^ *_2_*+σ^2^ *_3_*+*π* ^2^/3), using the REML-like estimate of σ^2^ *_A_* , with π^2^/3 being the variance of a standard logistic distribution, taking on the role of the environmental variance on the underlying liability scale. For the linear mixed model, heritabilities are estimated as 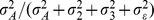 (model [EQ4]) or as 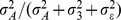 (Model [EQ5]). Using ASReml “pin” files, delta method estimates (i.e. first order Taylor series approximation) of the standard errors of the heritability estimates were also calculated.

#### 5) Maternal models

Maternal heritabilities and maternal environmental effects are estimates of the proportion of phenotypic variation due to the genetic and environmental developmental conditions which a dam provides for her offspring. They were examined in a separate ordinal (multi-threshold) analysis model which added additional random effects to the model above, a dam effect linked to a pedigree structure and a dam effect divorced from the pedigree structure, which modelled a maternal additive genetic component and a maternal environmental effect, respectively. However, the litter effect had to be removed from the models due to failure of convergence. This may be due to the increasing complexity and (partial) confounding of the litter effects and a combination of the maternal environmental effects and year effects which were also in the model, given that a dam only had one litter in a year.

## Results

### Completeness of Data Set

The completeness of the data set, expressed as the percentages of dogs for which scores were available, is presented in Table 2. The data set provides good coverage of dogs born in recent years, with scores available for the sires of 87.8% of puppies born in 2000–2005 and scores for the dams of 90.6% of puppies born in these years. Percentage coverage declines for dogs born earlier, although it is reasonable (greater than 50%) since 1991 and appears to be increasing. Care needs to be taken in making inferences about the use of hip dysplasia scoring by GSD breeders based on this table (although these numbers may be considered minimum values). Relative paucity of coverage in older date ranges could indicate fewer assessments for hip dysplasia but could equally reflect poorer submission rates or the possibility that older data have either been mislaid or were never made available electronically.

**Table 2:** Availability of British Veterinary Association Hip Trait scores for registered Australian German Shepherd Dogs.

	Year of birth					
	1976–1980	1981–1985	1986–1990	1991–1995	1996–2000	2001–2005
**% of born Puppies**						
with score available	0.2	2.2	5.7	8.8	11.2	10.7
with sire’s score available	1.3	6.5	28.4	50.8	61.4	87.8
with dam’s score available	0.4	4.8	24.2	53.1	65.2	90.6
**Sires**						
% unique sires with scores	0.4	3.8	16.7	34.6	54.5	85.6
**Dams**						
% unique dams with scores	0.3	4.5	21.0	47.3	62.6	87.9

In the most recent year-of-birth range (2001–2005), usage of the scheme appears to have been good for parents of Australian-born GSDs, with scores missing from only between 10–15% of parents. It is also worth noting that percentages worked out on a per-puppy basis have tended to be higher than when treating all sires and dams as equal regardless of the number of registered puppies that are produced. This suggests there is no evidence that unscored parents are being used more frequently than scored parents and may, indeed, be suggestive of the opposite. It is also possible that some of the sires and dams for which hip score data were not available have been assessed for hip dysplasia using other methods, or their scores were calculated but not recorded in either of the available data sets. The proportion of Australian-born-and-registered GSDs for which hip scores are available generally increased over time and is around 11% in the most recent year-of-birth ranges.

### Phenotypic Variation in BVAHTs

**Figure 1:**
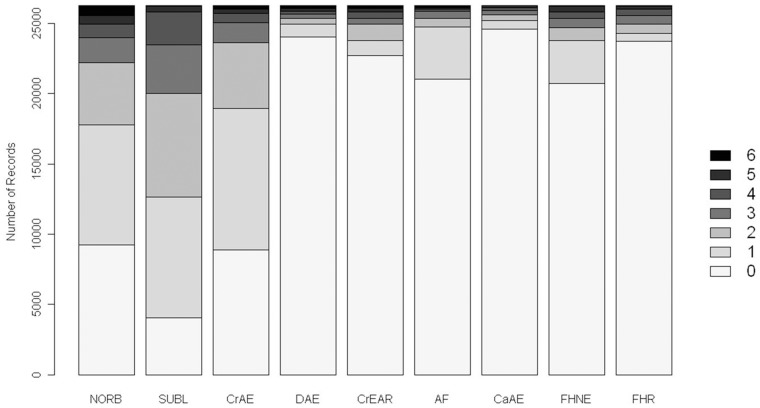
Distribution of British Veterinary Association Hip Trait scores in Australian German Shepherd Dogs.

**Figure 2:**
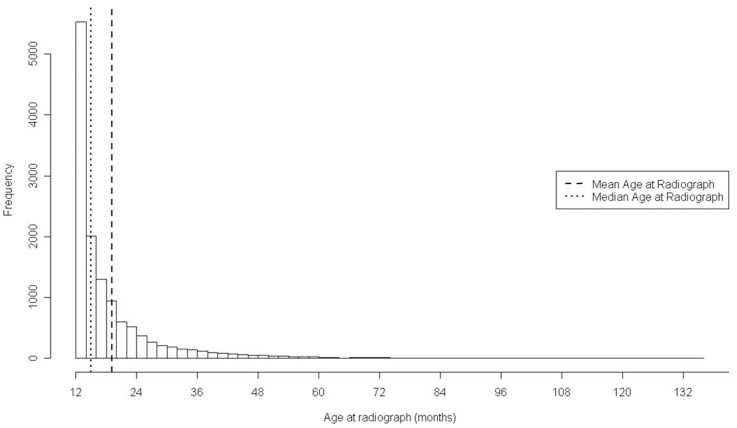
Reported age at time of radiograph for all 13,124 German Shepherd Dogs used in the present analyses.

The distribution of scores is illustrated in Figure 1. It is evident that the BVAHTs may be divided into two groups based upon their score distribution: Group 1 consisting of Norberg Angle (NORB), Subluxation (SUBL) and Cranial Actebular Edge (CrAE), in which there is substantial variation; and Group 2 consisting of Dorsal Acetabular Edge (DAE), Cranial Effective Acetabular Rim (CrEAR), Acetabular Fossa (AF), Caudal Acetabular Edge (CaAE), Femoral Head and Neck Exostosis (FHNE) and Femoral Head Remodelling (FHR) in which there is appreciably less variation. A similar finding was noted in a study of Gordon Setters from the United Kingdom where NORB, SUBL and CrAE accounted for 60% of the summed BVAHT [18]. Interestingly, Group 1 BVAHTs could be said to roughly correspond to traits most concerned with joint laxity and acetabular shallowing during development, whereas Group 2 BVAHTs roughly correspond to osteoarthritic changes. Given that the natural course of the disease is to worsen with respect to osteoarthritis over time, it would be expected for there to be fewer more-severe scores in the BVAHTs more associated with osteoarthritis, if animals were mostly scored at a relatively young age, before osteoarthritis has progressed to severe phenotypes. Figure 2 demonstrates that the age at which dogs in the sample were scored was young, on average, with a median (19 months) substantially less than the 24 months required for evaluation by some other organisations.

### Heritability

Heritability estimates for the BVAHTs obtained using ordinal models and LMMs for single BVAHTs are presented in Table 3. Generally, there was reasonable agreement between the LMM method and the more desirable ordinal method for most of the BVAHTs. While the point estimates varied between the methods, they were generally within one standard error, although CaAE estimates differed more substantially. All estimates represent an additive variation of sufficient proportion of the phenotypic variation to lead to a substantial response in a well designed selection program, indicating that selection using EBVs for these BVAHTs is likely to be successful in reducing BVAHT scores, given sufficient selection pressure. The LMM heritability estimate for the summed BVAHT was 0.30±0.02.

**Table 3:** Heritability estimates (*h^2^*), with standard errors (SE), of British Veterinary Association hip traits (BVAHTs) in Australian German Shepherd Dogs.

	Multi-threshold ordinal analysis		Log-transformed linear analysis (LMM)	
BVAHT	*h* ^2^	SE	*h* ^2^	SE
**Norberg Angle (NORB)**	0.23	0.02	0.23	0.02
**Subluxation (SUBL)**	0.23	0.02	0.18	0.01
**Cranial Acetabular Edge (CRAE)**	0.24	0.02	0.22	0.02
**Dorsal Acetabular Edge (DAE)**	0.14	0.02	0.14	0.02
**Cranial Effective Acetabular Rim (CrEAR)**	0.20	0.02	0.19	0.02
**Acetabular Fossa (AF)**	0.22	0.02	0.18	0.02
**Caudal Acetabular Edge (CAAE)**	0.20	0.03	0.25	0.02
**Femoral Head and Neck Exostosis (FHNE)**	0.21	0.02	0.22	0.02
**Femoral Head Remodelling (FHR)**	0.17	0.03	0.17	0.02
**Sum of BVAHTs**	NA	NA	0.30	0.02

**Figure 3:**
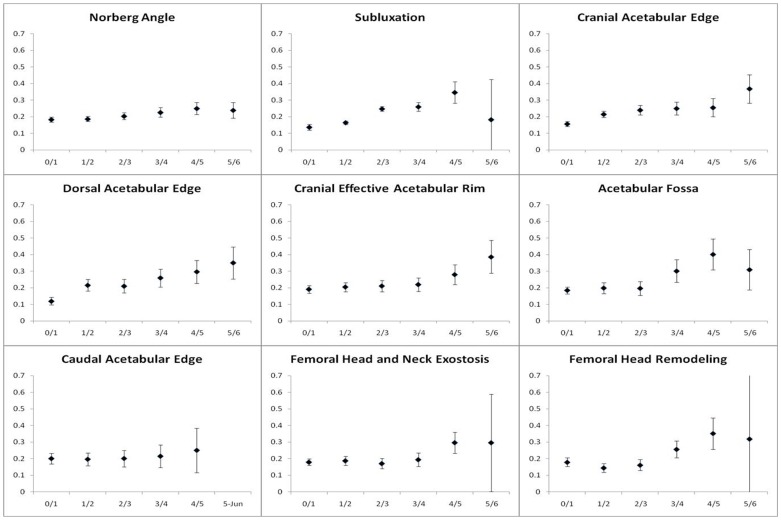
Heritability estimates of British Veterinary Association Canine Hip Dysplasia Phenotypes in a cohort of Australian German Shepherd Dogs when the ordinal phenotypes are expressed as binary outcomes.

Heritability estimates from binary models are presented in Figure 3. Standard errors associated with the higher cut-points tended to be larger due to a smaller proportion of dogs having increasingly higher scores (See Figure 1). For many of the BVAHTs there seems to be a trend toward increasing heritability as the cut-point increases. This could suggest either that non-genetic/environmental factors are more important in dogs with lower scores (and therefore that the additive heritable proportion of the variation is less) or possibly that the radiographic scoring is more repeatable for higher scores. Ultimately, the reason for this pattern is not understood and warrants further study.

### Age Effect

Age at scoring in months (see Figure 2) was included as a variable in the ordinal models. Based on Wald z-tests, increasing age resulted in significantly increasing scores for SUBL and all Group 2 traits (all P<0.05). While increases in osteoarthritis (Group 2) traits with age is not surprising, the increase in SUBL is somewhat surprising, given that remodelling may act to reduce laxity over time. It is possible that this effect is due to an apparent increase in the appearance of subluxation with age due to changes in the shape and relative position of other joint landmarks. Changes in NORB and CrAE over time were not statistically significant. The pattern observed in the LMM single-trait analysis was similar.

### Hip Effect

A previous paper [12] demonstrated that the genetic correlation between left and right hips for the BVAHTs is very high, but all traits except for AF and CaAE displayed marginal asymmetry, indicating a significant environmental hip effect. The left hip effects (compared to a reference right hip effect of zero) are presented in Table 4, along with standard errors. The left hip is associated with higher scores for all traits except NORB, and was significantly so for SUBL, CrAE, CrEAR, FHNE and FHR. The right hip was associated with significantly higher scores for NORB. As explained in the previous paper [12], these hip effects are not inconsistent with a high genetic correlation between hips.

**Table 4:** Hip and sex effects in British Veterinary Association Hip Trait (BVAHTs) scores in Australian German Shepherd Dogs for the multi-threshold ordinal analysis.

BVAHT	Left Hip Effect	(SE)	Male Sex Effect	(SE)
**Norberg Angle (NORB)**	−0.29	0.02	−0.58	0.04
**Subluxation (SUBL)**	0.10	0.02	−0.39	0.04
**Cranial Acetabular Edge (CRAE)**	0.12	0.02	−0.63	0.04
**Dorsal Acetabular Edge (DAE)**	0.09	0.05	−0.40	0.07
**Cranial Effective Acetabular Rim (CREAR)**	0.19	0.04	−0.62	0.06
**Acetabular Fossa (AF)**	0.03	0.04	−0.38	0.06
**Caudal Acetabular Edge (CAAE)**	0.06	0.05	−0.50	0.08
**Femoral Head and Neck Exostosis (FHNE)**	0.07	0.03	−0.34	0.05
**Femoral Head Remodelling (FHR)**	0.16	0.05	−0.44	0.07

### Sex Effect

Sex was also included as a fixed effect. Approximately 67% of records were from female dogs and 33% from male dogs. The size of the effects (male effect compared with a reference female effect of zero) and their standard errors are also presented in Table 4. For all of the BVAHTs, male sex was associated with significantly lower scores.

### Year-of-birth Effect

**Figure 4:**
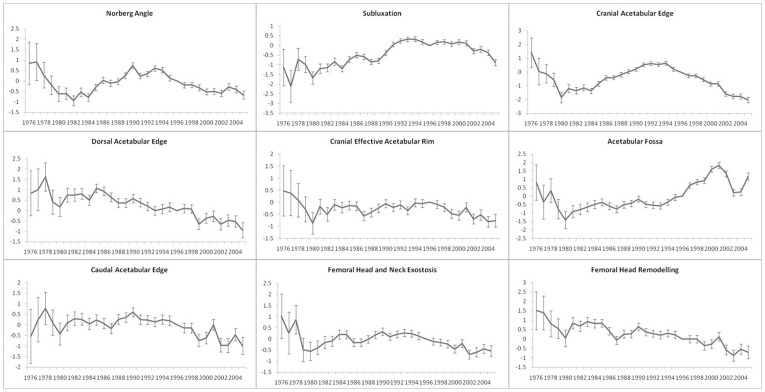
Non Genetic Year-of-Birth effects for British Veterinary Association Hip Trait Phenotypes in a population of Australian German Shepherd Dogs.

The year-of-birth effects were fitted as a factor (categorical term) rather than as a trend over time. These effects demonstrate differences in the sum total of all non-genetic effects from year to year. For many of the BVAHTs, the combined non-genetic effects appear to show a declining (favourable) trend over time (see Figure 4); for some, there is an apparent rise in the late 1980s and early 1990s. Overall, no particular trend in the combined non-genetic factors is observable across BVAHTs. For the majority of Group 2 (osteoarthritic) traits, the combined effect of non-genetic effects appears to demonstrate either a plateau or a slight fall, suggesting either no change, or a slight improvement in the combined non-genetic factors influencing these traits. It is worth noting that the majority of animals were scored when they were quite young and that more improvement from environmental management may become evident later in life.

For NORB and CRAE (Group 1 BHAHTs), the combined non-genetic factors follow a similar pattern, showing improvement up to the early 1980s, a worsening over the next decade, followed by improvement since. The trend in non-genetic effects for AF and SUBL is somewhat atypical. Given that these traits could be considered one of the better measures of joint laxity, which is suspected as the underlying lesion in hip dysplasia, it is pleasing to see an improvement in the combined effect of non-genetic factors since 2000. With the exception of AF, movements in the combined non-genetic effects have been in a favourable direction in recent times, suggesting that, overall, the present management strategies by breeders, owners and veterinarians are producing favourable results with regard to hip dysplasia.

### Litter Variance Component

Litter variance components from the ordinal (multi-threshold) trait analyses are shown in Table 5. All components-to-standard-error ratios exceeded 2 (range 2.86–7.65). NORB, SUBL and CrAE appear to have higher ratios than all the remaining traits. The percentage of the variance for which the litter effect accounted ranged between 5.82 and 8.70% and no pattern is evident.

**Table 5:** Litter variance component in British Veterinary Association Hip Trait (BVAHTs) scores in Australian German Shepherd Dogs for the multi-threshold ordinal analysis.

BVAHT	Litter Variance Component/Standard Error	% variance due to Litter Effect
Norberg Angle (NORB)	7.46	7.47
Subluxation (SUBL)	7.65	7.08
Cranial Acetabular Edge (CRAE)	6.26	6.57
Dorsal Acetabular Edge (DAE)	3.23	7.35
Cranial Effective Acetabular Rim (CREAR)	4.46	7.71
Acetabular Fossa (AF)	3.87	6.14
Caudal Acetabular Edge (CAAE)	3.31	8.70
Femoral Head and Neck Exostosis (FHNE)	4.06	5.82
Femoral Head Remodelling (FHR)	2.86	6.24

### Maternal Effect

As shown in Table 6, models including terms for a maternal mode of inheritance and a maternal environmental effect returned positive variance components for all traits except FHNE and FHR for which the maternal additive component was negative and of very small magnitude not significantly different from 0. Maternal environmental components were positive for all BVAHTs, and mostly in excess of twice their standard errors.

**Table 6:** Maternal genetic and environmental effects on British Veterinary Association Hip Trait (BVAHTs) scores in a cohort of Australian German Shepherd Dogs.

BVAHT	Maternal Additive Comp/SE	Maternal heritability	Maternal Environ. Comp/SE	% variance due to Maternal Env Comp
**Norberg Angle (NORB)**	2.44	0.03	3.08	2.64
**Subluxation (SUBL)**	2.57	0.03	3.63	3.13
**Cranial Acetabular Edge (CRAE)**	0.3	0.00	5.00	4.26
**Dorsal Acetabular Edge (DAE)**	0.47	0.01	1.45	2.56
**Cranial Effective Acetabular Rim (CREAR)**	1.07	0.01	3.38	4.47
**Acetabular Fossa (AF)**	0.71	0.01	3.78	4.93
**Caudal Acetabular Edge (CAAE)**	0.08	0.00	2.23	4.76
**Femoral Head and Neck Exostosis (FHNE)**	–	–	4.62	4.43
**Femoral Head Remodelling (FHR)**	–	–	2.09	2.86

## Discussion

This study was undertaken to investigate the presence of meaningful phenotypic variation in this population and to determine if this variation is sufficiently heritable to be amenable to selection pressure. While the ordinal models can give an estimate of model variance components relative to the residual variance ( π^2^/3) they cannot provide any information about the phenotypic variance on the underlying scale (variation in the precise quantitative amount of joint disease present). While linear models do provide an estimate of phenotypic variance on the observed scale (not shown), the phenotypes cannot be mapped onto the underlying scale, as it is not proven that the units of the observed scale (the BVAHT category numbers) are spaced equidistantly over the underlying scale. On the contrary, the units on the observed scale are arbitrary. A hypothetical observed scale with a different number or spacing of category numbers could give vastly different (and no more valid) phenotypic variance estimates to data which was identical on the underlying scale. However, although phenotypic variation cannot be quantified, Figure 1 clearly demonstrates that phenotypic variation is present and allows a comparison between the BVAHTs.

Although heritability estimates for CHD phenotypes are relatively abundant in the literature (see Table 1), heritability estimates of the BVAHTs are rarer [13], [15]–[18]. The presence of meaningful, heritable phenotypic variation in the BVAHTs demonstrates that BVA scores are amenable to improvement through selection. Selection refers, in effect, to identifying animals with superior alleles for a trait and breeding preferentially from these animals. This selective breeding aims to increase the frequency with which the superior alleles are found in the population (with a concomitant decrease in the frequency of less desirable alleles). The change in allele frequency leads to a change in BVA scores. The extent to which the change in allele frequency improves CHD-related welfare depends on the extent to which the alleles which determine a favourable BVA score also determine desirable welfare outcomes. Even setting aside the dog’s internal experience of CHD-related welfare, the relationship between BVA phenotypes and clinical hip dysplasia is virtually unstudied. However, Malm *et al*. [25] demonstrated an association between insurance claims for clinical hip dysplasia and a similar hip phenotype providing evidence of a genetic correlation between BVA -like phenotypes and clinical outcomes, and therefore a potential for selection for such phenotypes to modify the likelihood of clinical outcomes.

This paper presents heritability estimates of BVA hip traits in Australian German Shepherd Dogs obtained by taking into account the ordinal nature of the traits, using an ordinal logistic regression based REML-like mixed model. The models fitted also enabled evaluation of the importance of several fixed effects relating to right and left hip effects, year of the animal’s birth, its age at the time of radiography for scoring and its sex. Unfortunately data were not available for several other fixed effects which have been suggested as potential sources of variation for BVAHTs, including extent of sedation at the time of radiography [27] and identity of the radiograph evaluator. Had data on these effects been available, we would have been able to investigate the effect of these factors on BVAHT scores and, if necessary, adjust for any tendency for related animals to be more similar for these factors than unrelated animals which could result in over-estimation of heritability. Similarly, it would have been useful to be able to include the identity of the veterinarian taking the radiograph, or the veterinary practice in which the radiograph was taken, as a random effect, given that positioning has been reported in the literature [10] as a potentially significant source of variation. All CHD scoring protocols should include the recording of this information.

This study included nearly twice as many records from female dogs as from males, and for all nine BVAHTs female dogs tended to score more poorly. An effect of sex on BVAHT has been noted previously, but the more affected sex has been inconsistent across breeds [17]–[18]. In studies of other hip dysplasia phenotypes in the German Shepherd Dog, sex effects have been variable, with one study noting males were less affected [23] and another finding no significant difference between males and females [21]. The reason for male animals exhibiting lower BVAHT scores than females in this study is unclear. It is likely that a greater breeding selection pressure is placed upon males prior to submission of radiographs and that part of this selection pressure is toward a trait which is correlated with BVAHTs in some fashion, potentially related to the gait or ease of movement of the young dog. The pedigree file showing relationships between dogs for which there were data had similar sex proportions to those in the phenotype dataset, in that 31.3% of unique parents were male and 68.7% were female. If this pattern is consistent with the whole breeding population (i.e. individual sires father more than twice as many puppies as individual mothers), then there is no real indication of a sex-related submission bias. On the other hand, there may be a biological basis for this finding, given that the human analogue (developmental hip dysplasia) is substantially more common in female infants [28] and the potential for hormones to modulate CHD phenotypes has been postulated [29].

All nine BVAHTs are clearly heritable in our studied population: eight of the nine BVAHTs have a substantial heritability in the range 0.17–0.24; and the heritability of the other trait (DAE) is 0.14. Heritability estimates of the individual BVAHTs have been obtained from United Kingdom populations of Gordon Setters and Labrador Retrievers (Table 1) [15], [16], [18]. For both breeds the authors noted higher heritability estimates for NORB and SUBL than for other traits. Our results are somewhat similar, with the heritability of these two traits ranking equal second. Different breeds from different countries are not expected to have exactly the same heritability for a phenotype, as they are not expected to have exactly the same allele frequencies or the same set of non-genetic factors. The differences between the estimates from our studies and other studies are likely due to both the use of ordinal logistic regression in this study and to innate differences in the population. The heritability of the summed BVAHT scores was 0.30 in this study which is comparable to findings in UK Labradors, 0.34 (by a regression-based method) [16], and 0.35 (by a linear REML-based method) [13]. Heritability estimates for the summed scores in other breeds have varied from 0.20 to 0.75 (See Table 1).

Differences in heritability estimates between threshold and linear models for canine hip dysplasia have been compared previously, in a cohort of Estrela Mountain Dogs. Silvestre *et al*. [30] compared the use of a threshold model using a Bayesian approach, and an LMM using REML, in modelling a five-point ordinal categorical scale of hip dysplasia severity in use in Europe under the criteria of the Fédération Cynologic Internationale (FCI). The estimates of heritability obtained by the two methods were similar, which is consistent with the findings of this study [30]. Despite the similarity of the estimates from the two methods, there is no compelling reason to use an LMM for the analysis of data which are truly ordinal, now that an appropriate REML-like method is available for fitting ordinal models.

The accuracy of selection based on phenotypic selection is the square root of the heritability. Based on the ordinal model estimates, the accuracy for phenotypic (“performance”) selection based upon the BVAHT scores is between 0.40–0.52. This range is similar to the range of accuracies for the major dairy-cattle production traits, which have been improved so markedly by selection in recent decades.

Unlike the direct heritability estimates, the maternal heritability was very small in all cases. The relevant component divided by its standard error suggested statistical significance for only NORB and SUBL, and the absolute magnitude of the heritability for even these two traits was very small, suggesting there would be no great advantage in selecting for maternal protective traits for hip dysplasia within this population. Dietsche *et al*. [31] similarly reported only a small maternal heritability of a hip dysplasia trait, once a permanent maternal environmental effect was included.

The maternal environmental effect accounted for between 2.6% and 4.9% of the variance, and the relative size of the standard error suggested this may be statistically significant for all but one trait (DAE). A kennel effect was not included in the present analysis as there was no direct information about the environments in which dogs were raised and produced litters, having only the kennel prefix which reflects only into which kennel the dog was born. Therefore it is possible that identified maternal effects are in fact a proxy for an effect for some other non-genetic effect, rather than one specifically related to the mothering of the dam. Dietsche *et al*. [31] found maternal environmental effect accounting for around 10% of the phenotypic variation for another hip dysplasia trait. No additional variation was explained when they added a kennel effect to a model already including a maternal environmental effect.

Submission of radiographs radiographs for evaluation is voluntary, which creates a potential concern regarding submission bias among offspring. Additionally, obviously symptomatic animals may not be selected as potential parents, which could potentially create an evaluation bias among offspring. In later time-periods covered by the study, the proportion of hip scores among parents is high (see Table 2), but in earlier time-periods there is some potential for submission and evaluation biases among parents compared to all dogs scored, and there may be an additional bias against high scores in animals selected for radiography initially. Such selection would be expected to lower the phenotypic variation but also lower the variation in differences between families. Additionally, it appears that higher hip scores may be more heritable than lower hip scores (see Figure 3). From consideration of all these issues, the effect on heritability estimates of any submission or evaluation bias is not definitively predictable in sign or in magnitude. However, if there is a bias, its effect is likely to be small. The main purpose of this study was to evaluate whether heritability is sufficiently large to warrant selection in this population, and whether the use of EBVs is advisable. Particularly as the estimates reported in this paper are in broad agreement with other estimates in the literature, any bias (if present) has not compromised the conclusions reached.

Estimates of variation and of direct heritability by all methods were in the range that indicates that firstly, selection based upon these phenotypes can be effective and secondly, that development of EBVs which would allow the inclusion of phenotypic data from related animals could result in substantial improvements in accuracy of selection. The response to selection which can be expected from a genetic control scheme of this type depends on the phenotypic variation and heritability of the phenotype in the population, whether EBVs are used, the selection pressure which is applied by the end users of the scheme and how well the selection criterion (BVAHT scores) genetically correlates to the goal phenotype (also called breeding objective). Wilson *et al.* [32] argued that the goal phenotype is improved animal welfare. Consequently, the extent to which any phenotype used in a CHD control scheme genetically correlates to improved animal welfare is a key question. Our analysis suggests that with adequate selection pressure, BVAHT phenotype scores should improve over time, and would do so at a considerably faster rate if selection on EBVs replaces phenotype selection. The extent to which this improvement in BVAHT phenotypes would result in improved welfare is unfortunately unknown, but should be at least positive. Other types of radiographic evaluation may potentially have higher genetic correlations with welfare, and promising phenotypes should be evaluated in this population for variation, heritability and genetic correlation with welfare. Detailed discussion regarding the response to the selection which this population underwent over the time of the study is beyond the scope of this paper, but will be addressed in future work.

While selection based on BVAHT scores continues, the complex construction of the BVA phenotypes complicates the analysis. Assuming that the summed phenotypes are truly linear, as was probably the intention when the phenotypes were devised, simplifies the analysis by allowing the use of linear models. However, this approach makes several assumptions (discussed in the materials and methods) which may not be justified. We recommend that estimated breeding values for BVA hip traits be developed and implemented to increase the effectiveness of selection and that where possible, ordinal methods of analysis are utilised. Future papers will further explore the optimal methods for calculating EBVs for selection in this population.
